# Accessibility and use of urban green spaces, and cardiovascular health: findings from a Kaunas cohort study

**DOI:** 10.1186/1476-069X-13-20

**Published:** 2014-03-19

**Authors:** Abdonas Tamosiunas, Regina Grazuleviciene, Dalia Luksiene, Audrius Dedele, Regina Reklaitiene, Migle Baceviciene, Jone Vencloviene, Gailute Bernotiene, Ricardas Radisauskas, Vilija Malinauskiene, Egle Milinaviciene, Martin Bobak, Anne Peasey, Mark J Nieuwenhuijsen

**Affiliations:** 1 Lithuanian University of Health Sciences, Academy of Medicine, Institute of Cardiology, Kaunas, Lithuania; 2 Department of Environmental Sciences, Vytautas Magnus University, Kaunas, Lithuania; 3 Department of Epidemiology and Public Health, University College London, London, UK; 4 Center for Research in Environmental Epidemiology (CREAL), Barcelona, Spain; 5 Municipal Institute of Medical Research (IMIM-Hospital del Mar), Barcelona, Spain; 6 CIBER Epidemiologia y Salud Pública (CIBERESP), Barcelona, Spain

**Keywords:** Green spaces, Cardiovascular diseases, Risk factors

## Abstract

**Background:**

The aims of this study were to explore associations of the distance and use of urban green spaces with the prevalence of cardiovascular diseases (CVD) and its risk factors, and to evaluate the impact of the accessibility and use of green spaces on the incidence of CVD among the population of Kaunas city (Lithuania).

**Methods:**

We present the results from a Kaunas cohort study on the access to and use of green spaces, the association with cardiovascular risk factors and other health-related variables, and the risk of cardiovascular mortality and morbidity. A random sample of 5,112 individuals aged 45-72 years was screened in 2006-2008. During the mean 4.41 years follow-up, there were 83 deaths from CVD and 364 non-fatal cases of CVD among persons free from CHD and stroke at the baseline survey. Multivariate Cox proportional hazards regression models were used for data analysis.

**Results:**

We found that the distance from people’s residence to green spaces was not related to the prevalence of health-related variables. However, the prevalence of cardiovascular risk factors and the prevalence of diabetes mellitus were significantly lower among park users than among non-users. During the follow up, an increased risk of non-fatal and fatal CVD combined was observed for those who lived ≥629.61 m from green spaces (3rd tertile of distance to green space) (hazard ratio (HR) = 1.36), and the risk for non-fatal CVD–for those who lived ≥347.81 m (2nd and 3rd tertile) and were not park users (HR = 1.66) as compared to men and women who lived 347.8 m or less (1st tertile) from green space. Men living further away from parks (3rd tertile) had a higher risk of non-fatal and fatal CVD combined, compared to those living nearby (1st tertile) (HR = 1.51). Compared to park users living nearby (1st tertile), a statistically significantly increased risk of non-fatal CVD was observed for women who were not park users and living farther away from parks (2nd and 3rd tertile) (HR = 2.78).

**Conclusion:**

Our analysis suggests public health policies aimed at promoting healthy lifestyles in urban settings could produce cardiovascular benefits.

## Background

Cardiovascular diseases (CVD)–including coronary heart disease (CHD) and stroke–and cancer continue to be the leading causes of morbidity and mortality in most Western and Eastern countries [1-3]. The decline in the incidence and mortality from CVD and other non-communicable diseases lasting already several decades in most high-income countries is mainly attributable to lifestyle and other modifiable factors, including the reduction in smoking, control of high blood pressure and cholesterol levels, increasing physical activity, healthy nutrition habits, and other positive changes in cardiovascular risk factors [2,4].

There is mounting evidence that proximity to parks and other green spaces also has benefits for the health and health-related behavior of urban residents [5,6]. Possible causal mechanisms include the psychologically and physiologically restorative effects of contact with the natural environment, reduction of pollutants, and opportunities for social contacts and physical activity [7,8]. Some investigations of the associations between green space and human health have been based on evolutionary hypotheses, explaining that we as human beings have a genetic need for nature. By instinct, visiting green spaces makes us calmer and less stressed [9]. The impact of green spaces on health is also often explained by green space-obesity and green space-physical activity associations [10,11]. Green space has been related to lower CVD mortality, reduced stress, and better self-rated health, mental health, and cognitive functions [12-15].

In Lithuania, CVD incidence and mortality rates both among women and men are higher than in most European countries–especially in high-income Western European countries [16,17]. Epidemiological studies among random samples from rural and urban Lithuanian population found a high prevalence of most lifestyle-related and other modifiable risk factors of CVD [18,19]. In Lithuania–similarly as in other countries–the prognostic value of these risk factors on the incidence and mortality from CHD, stroke, and other non-communicable diseases has been studied for several decades, showing a significant impact of lifestyle and other risk factors [20,21]. Based on the literature and given the high levels of morbidity and mortality from CVD and unhealthy lifestyles in Lithuania, we hypothesized that accessibility and use of urban green spaces could be associated with health benefits for urban residents. The aims of this study, therefore, were the following: 1) to explore associations between the distance to and use of urban green spaces and the prevalence of known cardiovascular risk factors at baseline; 2) to evaluate the impact of the accessibility and use of green spaces on the incidence of CVD in a follow-up of middle-aged and elderly urban population.

## Methods

### Study area

The study area was Kaunas–the second largest city in Lithuania with a population of 360,637 in 2006. Kaunas is located at the confluence of two largest rivers of Lithuania–the Nemunas and the Neris, and near the Kaunas Reservoir–the largest body of water in Lithuania. The city covers 15,700 hectares, of which 8,329 hectares are covered by greenery (parks, groves, gardens, natural reserves, and agricultural areas). Our definition of “green space” included city parks larger than 1 ha, with 65% of land covered with trees. All the parks are open to the public, are located among residential homes or establishments, and near public transport lines, and offer some recreation opportunities (e.g., walking, jogging, rollerblading, physical training, or resting on the bench).

### Study cohort

This study was conducted as part of the PHENOTYPE project (Positive Health Effects of the Natural Outdoor Environment in Typical Populations in Different Regions in Europe) funded by the European Commission Seventh Framework Programme (http://www.phenotype.eu). The participants were men and women aged 45-72 years and living in Kaunas city, who were randomly selected to the HAPIEE (Health, Alcohol, and Psychosocial Factors in Eastern Europe) study from the National population register, and were stratified by sex and age; the study was performed in 2006-2008. In total, 5,112 responders (2,195 men and 2,917 women) participated in this survey. The response rate was 61.0%. The data collection during the HAPIEE study baseline survey included self-reported socio-demographic and health data, and also some measurements. The participants provided their residential addresses. We estimated the green space exposure for all responders who lived for at least one year at their current address.

### Measures and tests at baseline

At the baseline survey, measurements of blood pressure (BP), weight, and height, as well as laboratory analyses were conducted. BP was measured two times using a mercury sphygmomanometer and appropriately sized arm cuffs on the right arm. The initial measurement was performed on the right arm after five minutes of rest. After two minutes, the second measurement was performed. The Korotkoff phase 1 (the beginning of the sound) and the fifth phase of Korotkoff (the disappearance of the sound) was recorded as systolic and diastolic BP. The mean value of the two readings was used in the analysis. Hypertension was defined as mean systolic BP of at least 140 mm Hg or mean diastolic BP of at least 90 mm Hg, or both, and/or when the respondent had been taking drugs for high BP during the last two weeks. Weight and height were measured with a calibrated medical scale, and without shoes or heavy clothes. Body mass index (BMI) was calculated as weight in kilograms divided by the height in meters squared (kg/m^2^). Normal weight was defined as BMI <25.0 kg/m^2^, overweight–as BMI 25.0-29.9 kg/m^2^, and obesity–as BMI ≥30.0 kg/m^2^.

Cognitive function was assessed using a battery of five standard tasks. Immediate and delayed verbal memory was assessed using a 10-word learning test. Semantic verbal fluency was examined by asking the participants to name as many animals as possible within 1 minute. Speed and concentration were tested by asking the participants to cross out as many target letters as possible within 1 minute. Numerical ability was assessed using four questions involving simple calculations based on everyday situations. Because the scoring of each cognitive test varied, test scores were standardized to give a mean of 0 and a standard deviation of 1 (z-scores). Scores representing the composite score of cognitive function were obtained by averaging z-scores in all tests. Low cognitive function was defined using the composite score of cognitive function. To control for the effect of age and education on the scores, the subjects were stratified into six age groups and five levels of education. The participants who scored 1 SD or more below their age and education-specific means of the composite score of cognitive function were ascribable to a low cognitive function group [22,23].

### Laboratory analyses

Biochemical analyses were conducted on samples taken on an empty stomach. Serum lipid concentrations were measured using the conventional enzymatic technique. The subjects were classified into three groups according to their total cholesterol level: normal (< 5.2 mmol/L), intermediate (5.2-6.19 mmol/L), and increased (equal to or above 6.2 mmol/L or more). Glucose concentration in capillary blood was evaluated by using an individual glucometer “Glucotrend” [24]. Normal glucose level was defined as fasting glucose <5.55 mmol/L, intermediate–as glucose level 5.55-6.98 mmol/L, and increased–as glucose level equal to or above 6.99 mmol/L.

### Variables obtained using the questionnaire

The standard questionnaire included questions regarding the respondents’ age, education, smoking status, physical activity, use of green space, time spent in the city parks per week, self-rated health and quality of life, etc. Education was classified into four education levels: primary, vocational or college, secondary, and university. Smoking habits were assessed according to the current smoking status. The respondents were classified into three groups: current smokers, former smokers, and never-smokers. A subject who smoked at least one cigarette per day was classified as a current smoker. Physical activity was determined by adding up the average time spent per week on walking, moderate and hard work, gardening, and other physical activities during leisure time in winter and summer. The respondents were categorized into two groups according to their physical activity in leisure time: active (10 and more hours/week), and inactive (<10 hours/week).

Symptoms of depression were measured using the 10-item Center for Epidemiologic Studies Depression Scale (CES-D 10) [25]. The subjects were asked to evaluate the presence of 10 depression symptoms during the past week on a two-point scale: yes or no. Each symptom was scored from 1 (yes) to 0 (no), resulting in total score of 0 to 10. The subjects with CES-D 10 scores of 4 or more were classified as having symptoms of depression.

### Definitions of CVD and diabetes mellitus

CHD was determined by: 1) a documented history of myocardial infarction (MI) and (or) ischemic changes on electrocardiogram (ECG) coded by the Minnesota codes (MC) 1-1 or 1-2 [26]; 2) angina pectoris was defined by G. Rose’s questionnaire (without MI and (or) MC 1-1 or 1-2; 3) [27]; ECG findings by MC 1-3, 4-1, 4-2, 4-3, 5-1, 5-2, 5-3, 6-1, 6-2, 7-1, 8-3 (without MI and (or) MC 1-1, 1-2 and without angina pectoris). Diabetes mellitus was determined according to the answers of the respondents to the question “Has a doctor ever told you that you have diabetes?” and/or fasting glucose level ≥7.8 mmol/L. Stroke was determined using the question “Has a doctor ever told you that you have had a stroke?”

### Follow-up of the cohort

The participants of the surveys were followed-up from the beginning of the baseline survey until December 31, 2011 by the regional CHD, stroke, and mortality registers (the mean duration of follow-up was 4.41 ± 0.94 years). The proportion of the participants lost to follow-up was 0.86% (N = 44). People who were lost to follow-up were censored at their last date of contact. Analysis of CVD mortality and morbidity was performed. The CVD mortality group consisted of deaths from CVD (codes of the 10th International Classification of Diseases (ICD) I00-I99). During the same period of follow-up, all incident cases of non-fatal CHD (acute myocardial infarction and unstable angina pectoris) and stroke were also registered. All fatal and non-fatal CVD cases were included into the “total CVD” group. During the follow-up, there were 83 deaths from CVD and 364 non-fatal cases of CVD among persons free from CHD and stroke at the baseline survey.

### Assessment of green space exposure

Spatial land cover data sets for Kaunas city were obtained from the municipality, and were processed using ArcGIS 10 software to produce the classification of green space exposure. Our definition of “green space” included city parks larger than 1 hectare. All self-reported home addresses of the survey responders were geocoded using the SAS/GIS geocoding software, and the distance to the nearest city park was estimated. To assess the effect of the living environment on cardiovascular risk factors and the prevalence of CVD, the study subjects were spatially linked to the three distance categories of green space exposure based on tertiles of the distance to the nearest green space (the 1st tertile–≤347.8 m (high); the 2nd tertile–347.81–629.6 m (moderate); and the 3rd tertile–≥629.61 m (low)). The responders’ home locations were mapped using ArcGIS 10, and were combined with a comprehensive GIS database of green space characteristics.

### Statistical analysis

SPSS version 13.4 software for Windows was used for statistical analysis. Distributions of the study cohort characteristics were tabulated by the distance from green spaces and by the use of city parks. Descriptive statistics (adjusted by age) were calculated and included into the variables of the analysis. All data were age-adjusted to the Kaunas population census of 2006. Coefficients 1.62 (age group 45-54 years), 0.97 (age group 55-64 years), and 0.75 (age group 65 years and older) were used in calculating the age-adjusted prevalence and means. Coefficients were calculated by dividing the coefficient for each age group by the sum of coefficients for three age groups. Chi-square (χ^2^) tests were used for testing the association of various variables with exposure to green spaces and the use of city parks. P < 0.05 was defined as statistically significant. Odds ratios (OR) and 95% confidence intervals (CI) of park use in relation to CVD risk factors and other variables, and OR and 95% CI of prevalent chronic diseases in association with the distance to the nearest green space were calculated using multivariate logistic regression models. We obtained estimates of the hazard ratio (HR) and 95% CI using the multivariate Cox proportional hazards regression for the incidence of non-fatal CVD (the incidence of acute myocardial infarction, unstable angina pectoris, and stroke) and the incidence of total CVD (all non-fatal CVD and fatal cases of CVD). Two multivariate Cox proportional hazard regression models were introduced. The first model included age, sex, cardiovascular risk factors (smoking, arterial hypertension, low physical activity, high total cholesterol level, high glucose level, overweight, and obesity), diabetes mellitus, low cognitive function, symptoms of depression, self-rated health, quality of life, exposure to green spaces, and the use of city parks in relation the risk of non-fatal CVD. The second model includes age, sex, the same cardiovascular risk factors, diabetes mellitus, low cognitive function, symptoms of depression, self-rated health, quality of life, and exposure to green spaces in relation to the risk of hard CVD. The covariates were selected a priori. All models were conducted for men, women, and for both men and women free from CHD and stroke at baseline survey.

## Results

### Baseline

High age-standardized rates of arterial hypertension, overweight, obesity, and hypercholesterolemia were observed among the participants (Table 1). There was no statistically significant difference in the distribution by sex in relation to the accessibility of green spaces (Table 2). The proportion of the participants aged 45-54 years in the 1st tertile of the distance to green spaces was significantly lower (24.3% and 26.9%), and proportion of the subjects aged 65 years or older was significantly higher (37.3% and 33.0%), compared to the 3rd tertile. The age-standardized mean age in the 3rd tertile was significantly lower, compared to the 1st tertile (57.9 ± 7.62 years and 58.5 ± 7.96 years) (Additional file 1: Table S1). The prevalence of all known cardiovascular risk factors (with exception of smoking and chronic non-communicable diseases) was unrelated to the access to green spaces (Additional file 1: Tables S1, S2, and S3).

**Table 1:** Selected characteristics of the study participants

Characteristic*	
Age at entry, mean ± SD, years	60.4 ± 7.49
Systolic BP, mean ± SD, mmHg	141.1 ± 21.8
Diastolic BP, mean ± SD, mmHg	90.1 ± 12.3
Total serum cholesterol, mean ± SD, mmol/L	5.98 ± 1.14
Fasting glucose, mean ± SD, mmol/L	5.80 ± 1.18
BMI, mean ± SD, kg/m^2^	29.3 ± 5.20
Proportion of men (%)	2163 (43.3)
Current smokers (%)	852 (16.8)
Leisure-time physical inactivity (%)	1176 (23.8)
Arterial hypertension (%)	3352 (67.6)
Overweight (%)	1936 (39.3)
Obesity (%)	1996 (40.5)
Fasting glucose level 7.0 mmol/L or more (%)	432 (8.9)
Total serum cholesterol 6.2 mmol/L or more (%)	1947 (39.5)
Prevalence of CHD (%)	892 (18.0)
Prevalence of stroke (%)	75 (1.5)
Prevalence of diabetes mellitus (%)	354 (7.2)
Green space users (%)	2543 (49.7)

BP–blood pressure. *all prevalence rates and means are age-standardized.

BMI–body mass index.

CHD–coronary heart disease.

SD–standard deviation.

**Table 2:** Distribution (%) of urban population aged 45-72 years according to the distance to green spaces

	**Distance to green spaces**			**Total**
	**1st tertile of the distance to green spaces N = 1694**	**2nd tertile of the distance to green spaces N = 1702**	**3rd tertile of the distance to green spaces N = 1716**	
**Sex**				
Men, n = 2163	43.8	43.6	42.3	43.3
Women, n = 2837	56.2	56.4	57.7	56.7
**Total,** n = 5000	100.0	100.0	100.0	100.0
**Age, years**				
45-54, n = 1237	24.3	23.0	26.9*##	24.7
55-64, n = 2041	38.5##	43.9**	40.1#	40.8
≥ 65, n = 1722	37.3##	33.0**	33.0**	34.4
**Total,** n = 5000	100.0	100.0	100.0	100.0
**Education**				
Primary, n = 268	5.0	4.0	3.4	4.1
Vocational and				
college, n = 1607	31.9	32.9	31.7	32.2
Secondary, n = 1286	25.8	26.9	27.9	26.9
University, n = 1789	37.3	36.2	37.0	36.8
**Total,** n = 4950	100.0	100.0	100.0	100.0

χ^2^ = 0.88, p = 0.64–for sex; χ^2^ = 17.6, p = 0.001–for age; χ^2^ = 7.88, p = 0.25–for education. *p < 0.05, **p < 0.01, as compared to the 1st tertile; #p < 0.05, ##p < 0.01 as compared to the 2nd tertile (proportions were compared using the Z test).

Distance to green space: the 1st tertile–≤347.8 m (high); the 2nd tertile–347.81–629.6 m (moderate); and the 3rd tertile–≥629.61 m (low).

The proportion of park users among people in the 1st tertile of the distance to green spaces was statistically significantly higher, compared to that among people from the 2nd and the 3rd tertiles: 55.0%, 50.3% and 44.1% respectively, p < 0.01) (Table 3). Compared to non-users, park users were less likely to smoke regularly, be obese and physically inactive, to have high levels of fasting glucose (≥ 7.0 mmol/L), to be of very poor or poor self-rated health and quality of life, and had a lower prevalence of diabetes mellitus. The odds of park use were significantly lower among regular smokers, obese people, those physically inactive during leisure time, people with medium or high fasting glucose levels, those self-rating their health and quality of life as average, poor, or very poor, and persons with diabetes mellitus, compared to persons with normal levels of the indicated risk factors, those rating their health and quality of life as good or very good, and those without diabetes mellitus (Table 4).

**Table 3:** Age-standardized distribution (%) of urban population aged 45-72 years by park use according to the distance to green spaces (parks)

**Visiting of parks**	**Distance to green spaces in tertiles**			**χ**^**2 **^**and p value**
	**1st tertile**	**2nd tertile**	**3rd tertile**	
**Men**	N = 742	N = 734	N = 997	7.67, p = 0.022
Yes	52.2	48.9	44.9**	
No	47.8	51.1	55.1	
**Women**	N = 952	N = 968	N = 919	37.0, p < 0.001
Yes	57.1#	51.3*	43.4***###	
No	42.9	48.7	56.6	
**Men and women**	N = 1694	N = 1702	N = 1716	40.8, p < 0.001
Yes	55.0##	50.3**	44.1***###	
No	45.0	49.7	55.9	

*p < 0.05, **p < 0.01, ***p < 0.001 as compared to the 1st tertile; #p < 0.05, ##p < 0.01, ###p < 0.001 as compared to the 2nd tertile.

All data were age-adjusted to the Kaunas population census of 2006 (details in section Methods “Statistical analysis”). Distance to green spaces: the 1st tertile–≤347.8 m (high); the 2nd tertile–347.81–629.6 m (moderate); and the 3rd tertile–≥629.61 m (low).

**Table 4:** Distribution (%) of prevalent health-related variables in urban population aged 45-72 years according to self-reported park use

**Risk factors**	**Self-reported park use**		**Odds ratio (OR)# of park use**	**χ**^**2 **^**and p value**
	**Yes**	**No**	**OR (95% CI)**	
	**N = 2543**	**N = 2569**		
*Smoking*				14.6, p = 0.001
Regular	17.4	21.0**	0.82 (0.69-0.97)	
Ex-smoker	16.7	18.0	0.93 (0.79-1.09)	
Never	65.9	61.0***	1 (Reference)	
*Total cholesterol, mmol/L*				0.72, p = 0.698
<5.2	24.7	25.7	1 (Reference)	
5.2-6.19	36.4	35.8	1.08 (0.93-1.25)	
≥6.2	38.9	38.5	1.06 (0.92-1.22)	
*BMI, kg/m*^*2*^				14.2, p = 0.001
<25.0	23.0	21.0	1 (Reference)	
25.0-29.9	40.9	37.7*	0.99 (0.85-1.16)	
≥30.0	36.2	41.3***	0.75 (0.64-0.88)	
*Arterial hypertension, mmHg*				0.84, p = 0.360
Yes	64.2	65.5	0.92 (0.81-1.04)	
No	35.8	34.5	1 (Reference)	
*Leisure-time physical activity*				21.2, p < 0.001
Active	78.3	72.8	1 (Reference)	
Inactive	21.7	27.2***	0.74 (0.64-0.84)	
*Fasting glucose level, mmol/L*				10.9, p = 0.004
<5.55	48.0	44.2**	1 (Reference)	
5.55-6.99	44.7	46.5	0.86 (0.76-0.96)	
≥7.0	7.3	9.3*	0.67 (0.55-0.83)	
*Self-rated health*				6.63, p = 0.036
Very poor and poor	11.3	13.1*	0.69 (0.56-0.83)	
Average	57.9	58.6	0.86 (0.76-0.99)	
Very good and good	30.8	28.3*	1 (Reference)	
*Quality of life*				8.82, p = 0.012
Very poor and poor	3.0	4.1*	0.63 (0.46-0.85)	
Average	44.7	47.1	0.88 (0.78-0.99)	
Very good and good	52.3	48.8*	1 (Reference)	
*Coronary heart disease*				0.05, p = 0.828
No	84.0	83.8	1 (Reference)	
Yes	16.0	16.2	0.91 (0.78-1.06)	
*Stroke*				0.18, p = 0.675
No	98.5	98.7	1 (Reference)	
Yes	1.5	1.3	1.06 (0.67-1.69)	
*Diabetes mellitus*				4.65, p = 0.031
No	94.3	92.8	1 (Reference)	
Yes	5.7	7.2*	0.72 (0.58-0.90)	

BMI–body mass index, CI–confidence interval, **#** adjusted by: age, sex, and education. *p < 0.05, **p < 0.01, ***p < 0.001, compared to “Yes”.

Arterial hypertension “Yes” = mean systolic blood pressure (BP) of at least 140 mm Hg or mean diastolic BP of at least 90 mm Hg, or both, and/or that respondent had been taking antihypertensive drugs during the last two weeks. Arterial hypertension “No” = systolic BP < 140 and diastolic BP <90 mm Hg.

### Follow-up

The risk of total CVD among Kaunas city population was statistically significantly related to the distance to green spaces; the hazard ratio among persons from the 3rd tertile of the distance to green spaces was 1.36 (95% CI 1.03-1.80), compared to persons from the 1st tertile (Table 5). The increased risk of total CVD in relation to the accessibility of green spaces was only statistically significant among men, but not among women; the hazard ratio among men from the 3rd tertile was 1.51 (95% CI 1.04-2.19), compared to those from the 1st tertile. The risk of non-fatal CVD among non-users living farther away than 347.81 m (the 2nd and the 3rd tertile) was statistically significantly increased (HR = 1.66, 95% CI 1.01-2.73), compared to users nearby. A statistically significantly increased risk of non-fatal CVD was observed in women who were not park users and were from the 2nd and the 3rd tertiles of the distance to green spaces, compared to the reference group (women from the 1st tertile and park users) (HR = 2.78, 95% CI 1.16-6.70), but this trend was not observed in men. Although the directions were similar, we found no statistically significant results with indicators of the European guidelines (0.5 hectare within 300 m).

**Table 5:** Adjusted hazard ratio (HR) of distance to green spaces and park use among Kaunas middle-aged and elderly population and the risk of CVD

**Analyzed health-related factors**	Risk of total CVD*	Risk of non-fatal CVD*
	**HR (95% CI)**	**HR (95% CI)**
*Men and women*		
*Distance to green spaces*		
1st tertile	1 (Reference)	--
2nd tertile	1.20 (0.90-1.61)	--
3rd tertile	1.36 (1.03-1.80)	--
*Distance to green spaces and park use*		
1st tertile x user	--	1 (Reference)
1st tertile x non-user	--	1.50 (0.83-2.72)
2nd and 3rd tertile x user	--	1.58 (0.95-2.63)
2nd and 3rd tertile x non-user	--	1.66 (1.01-2.73)
*Men*		
*Distance to green spaces*		
1st tertile	1 (Reference)	--
2nd tertile	1.38 (0.94-2.03)	--
3rd tertile	1.51 (1.04-2.19)	--.
*Distance to green spaces and park use*		
1st tertile x user	--	1 (Reference)
1st tertile x non-user	--	0.96 (0.44-2.12)
2nd and 3rd tertile x user	--	1.47 (0.80-2.70)
2nd and 3rd tertile x non-user	--	1.17 (0.63-2.18)
*Women*		
*Distance to green spaces*		
1st tertile	1 (Reference)	--
2nd tertile	1.06 (0.67-1.66)	--
3rd tertile	1.22 (0.79-1.89)	--.
*Distance to green spaces and park use*		
1st tertile x user	--	1 (Reference)
1st tertile x non-user	--	1.80 (0.71 4.56)
2nd and 3rd tertile x user	--	2.61 (0.97-7.02)
2nd and 3rd tertile x non-user	--k	2.78 (1.16-6.70)

CVD–cardiovascular diseases, CI–confidence interval. n.s.–not significant. -- - not included into the model. *adjusted by: age, sex, education, smoking, arterial hypertension, physical activity, total cholesterol level, fasting glucose level, body mass index, diabetes mellitus, cognitive function, symptoms of depression, self-rated health, and quality of life. Non-fatal CVD–all incident cases of non-fatal acute myocardial infarction, unstable angina pectoris, or stroke. Total CVD–all fatal and non-fatal CVD cases. Distance to green space: the 1st tertile–≤347.8 m (high); the 2nd tertile–347.81–629.6 m (moderate); and the 3rd tertile–≥629.61 m (low).

## Discussion

In this study, we found no or little association between objectively measured access to green spaces and known cardiovascular risk factors and the prevalence of most common chronic non-communicable diseases at baseline, but we found associations with the use of green space. Also, we found statistically significant associations between objectively measured green space measures and fatal and non-fatal CVD in the follow-up after adjusting for a range of other risk factors, with some apparent differences between men and women.

The results from the baseline data of our study suggest that objectively measured access to green space in people’s environment has little or no influence on people’s levels of known cardiovascular risk factors or the prevalence of most common chronic non-communicable diseases, such as CHD, stroke, and diabetes mellitus. No significant relationship was found between the distance to green spaces and the prevalence of arterial hypertension, hypercholesterolemia, hyperglycemia, excess body weight, leisure physical inactivity, low cognitive function, and symptoms of depression at baseline. Our results are in accordance with other studies in which no significant association was observed between exposure to green spaces and levels of physical activity, and the prevalence of obesity or overweight [28,6,29]. However, many studies and literature reviews have reported that a more natural living environment is related to better self-rated health and lower levels of some objectively measured or self-reported health factors, and morbidity and mortality rates [30,19,11]. The discrepancy in results may be due to, for example, different accessibility of green space measures, the studied populations, study designs, population sizes, and the contribution of other risk factors. There are numerous possible explanations for why we did not find any statistically significant differences in the prevalence of chronic non-communicable diseases and risk factors in relation to the distance to green spaces. Firstly, it may because some socio-economic characteristics of the study participants that were closely related to their health indicators were not analyzed. Although we controlled for education in this study, we did not take account of other socio-economic variables as, for example, income and socioeconomic position. Kaunas city areas could differ in their inhabitants’ income. City areas near green spaces are surrounded by private one- or two-flat houses. Residents in these areas usually have higher income and their own domestic gardens, and therefore activities in parks and other green spaces of the city are less important to them [13]. In our study, the definition of physical activity during leisure time included not only activity most likely to be undertaken in city green spaces, but also the overall physical activity, which might also suggest why we did not find any statistically significant difference in the prevalence of physical inactivity among study participants from the 3rd and the 1st tertiles of the distance to green spaces. People with low socio-economic status are less likely to exercise than those with a high socioeconomic position–partially because the environments in their neighborhood are less conducive to this [31,32]–and also less safe. It is suggested that not access *per se*, but access to attractive large open spaces or green spaces is what matters in the association of physical activity and green spaces [33]. The quality of city green spaces–including recreational facilities–has not been evaluated in our study: we did not have details on the specific features of each green space. A valuable extension of this work would provide a better understanding of which features might be acting to encourage the use of green spaces, as this insight could be used by city planners in the design of new green spaces and the regeneration of the old ones.

Although many studies have shown that the objectively measured access to green spaces in the urban populations could enhance health or healthy behaviors, only a few evaluated health-related factors in relation to the frequency of green space use [34,11]. In our study, the prevalence of green space use significantly declined with increasing distance from the green space; this was observed both among men and women. Similar findings were presented by Coombes et al. from the survey of 6,821 adults in the urban settings in the U.K. [11]. In the study of 4,899 Dutch people, no relationship was found between the amount of green space and whether or not people participated in sport activities and the number of minutes spend on sport activities [35]. In our study, the prevalence of self-reported or measured lifestyle-related (regular smoking, leisure physical inactivity) and biological (high levels of fasting glucose and obesity) cardiovascular risk factors was significantly lower among green space users than among non-users. Other studies also found healthier behavior and better physical or mental health among green space users. The results from the 2005 Danish Health Interview Survey showed that the more often the respondents visited green spaces, the less stress they experienced. Furthermore, the results indicated that the longer the distance was from the respondents’ homes to the nearest green space, the more stress they experienced [36,37]. The Health Survey of England found that people living in the greenest areas of England were more likely to use green spaces to achieve the recommended amounts of physical activity, both before and after adjustment for individual and environmental variables [37].

We also examined the association between the distance to green spaces, the use of these green spaces, and the combination of morbidity and mortality from CVD in this well-defined cohort, taking into account many known cardiovascular risk factors. The follow-up period was rather short–the mean duration of the follow-up was 4.41 ± 0.94 years. Therefore, as end-point, we used the incidence of non-fatal CVD (pooled cases of unstable angina pectoris, acute myocardial infarction, or stroke) and total CVD (pooled non-fatal CVD and cases of death from CVD) among persons free from CHD and stroke at baseline survey. An increase in the distance to green spaces was related to a higher risk of the incidence of total CVD adjusted for other cardiovascular risk factors and other health-related variables; this was observed both among men and the whole cohort. Compared to park users, statistically significantly increased risk of non-fatal CVD was observed for women who were not park users–but not for men. A number of studies examined all-cause and cause-specific mortality and morbidity in relation to exposure and use of green spaces [38,12,40,35,14]. In a cohort study of 575,000 adults in Ontario, Canada, findings showed that individuals who lived in areas with more green space had lower cause-specific mortality rates. The inverse mortality associations persisted after adjusting for a variety of socio-demographic and neighborhood characteristics. Rate ratios, however, were not adjusted for lifestyle or biological cardiovascular factors [12]. A cross-sectional study in the U.K. showed that inequality in all-cause and circulatory disease mortality related to income deprivation was lower in populations who lived in the greenest areas, compared to those who had less exposure to green space [41]. However, an observational study of a population of 1,546,405 living in small urban areas in New Zealand found no evidence that green space influenced cardiovascular mortality [39]. An ecological cross-sectional study in the largest US cities also concluded that there was no association between greenness and mortality from heart disease, diabetes, lung cancer or automobile accidents. Mortality from all causes was even statistically significantly higher in greener cities [40]. We found that associations between the exposure to and the use of green spaces and CVD mortality and morbidity differed among the male and female participants of the study. It could be partially explained by various social and physical characteristics of the neighborhood being more strongly associated with women’s than with men’s health [38]. Another possible explanation is that women and men may experience and utilize green spaces in different ways. Women are often under-represented in public parks, and are less likely to engage in vigorous physical activity there [34]. In our study, a larger proportion of women were park users, compared to men. We suggest that women visited parks more frequently and spent more time in the green spaces than men did because they are more likely to be supervising children and grand-children, and working part-time. This could partially explain why green space availability was more important for women’s health.

This is the first large epidemiological study in Central and Eastern Europe investigating the relation between the exposure to green spaces and the prevalence of cardiovascular risk factors and the incidence of CVD. The strength of this study is its cohort study design, the objective measures of the individual cohort members’ living distance to green spaces, the adjustment for many known risk factors, and measures for the actual use of green space. In addition to that, we used not only self-reported, but also objectively measured health-related variables. Data on the incidence of CVD were obtained from the regional registers of CHD and stroke with excellent ascertainment. Our study had its limitations. First, we investigated the distance to the available green spaces for each cohort member, but did not consider the type and the quality of the green space. The quality of the green space could be a substantial determinant of the use of the green space and activity within it [33]. We did not consider road, railway networks, or other holdups between the cohort members’ living place and green places either. This means that we could have included green spaces that are hard to reach because of natural or physical boundaries. Second, the cohort follow-up period was rather short, and therefore we may have lacked the statistical power due to the small number of the incident CVD cases. Third, the response rate at the baseline survey was not very high. This is a common problem in most epidemiological studies; in our study, non-responders were more likely to be male, younger, less educated, and less healthy than responders [42]. Finally, the territory of our study was limited to the second-largest Lithuanian city (Kaunas city). Further studies are needed to determine whether conclusions of our study can be generalized to other Lithuanian (at least) urban settings.

## Conclusions

This study found that the distance to green spaces was unrelated to the prevalence of CVD, known cardiovascular risk factors, or other health related variables. However, the prevalence of self-reported or measured lifestyle-related (regular smoking, and leisure physical inactivity) and biological (high levels of fasting glucose, and obesity) cardiovascular risk factors and the prevalence of diabetes mellitus was significantly lower among park users than among non-users. An increase in the distance from the living place to green spaces was related to a higher risk of the incidence of total CVD after adjustment for other cardiovascular risk factors and other health-related variables; this trend was observed both among men and the whole cohort. Compared to park users living at close distance to green spaces, a statistically significantly increased risk of non-fatal CVD was observed for the whole population and women who were not park users and living farther away from green spaces. Our study contributes to the evidence that green spaces can help fight some major public health threats in the society. Our analysis also suggests public health policies aimed at promoting healthy lifestyles in urban settings. The provision of green spaces on the neighborhood scale should be balanced by attention to the density of the city population, connectivity, land use, transportation infrastructure, and other city-scale predictors of good health.

## Abbreviations

BMI: Body mass index; BP: Blood pressure; CES-D 10: 10-item Center for Epidemiologic Studies Depression Scale; CHD: Coronary heart disease; CI: Confidence interval; CVD: Cardiovascular diseases; ECG: Electrocardiogram; HDL: High-density lipoprotein; HR: Hazard ratio; LDL: Low-density lipoprotein; MC: Minnesota codes; MI: Myocardial infarction; SD: Standard deviation; U.K.: United Kingdom; US: United States.

## Competing interests

The authors declare that they have no competing interests.

## Authors’ contributions

AT contributed to writing the manuscript, the study concept and design, and the analysis and interpretation of the data. RG and MJN contributed to writing the manuscript and the analysis and interpretation of the data. MBo and AP contributed to the study concept and design, and the analysis and interpretation of the data. RRe, MBa, GB, RRa, and VM contributed to writing the manuscript. DL, AD, and EM contributed to writing the manuscript, the study concept and design, and the analysis and interpretation of the data. JV contributed to the interpretation of the data. All authors read and approved the final manuscript.

## Supplementary Material

Additional file 1: Table S1 Age-standardized means (± SD) of the health-related variables in urban population aged 45-72 years according to the distance to green spaces. **Table S2.** Age-standardized distribution (%) of cardiovascular risk factors in urban population aged 45-72 years according to the distance to green spaces. **Table S3.** Age-standardized distribution (%) and odds ratios (OR) of chronic diseases in urban population aged 45-72 years according to the distance to green spaces. Click here for file
