# Supplementary material for: Accessibility and use of urban green spaces, and cardiovascular health: findings from a Kaunas cohort study
Source: Environ Health. 2014 Mar 19;13:20. doi: 10.1186/1476-069X-13-20 (PMC4000006; doi:10.1186/1476-069X-13-20)
Supplement: Additional file 1: Table S1 — Age-standardized means (± SD) of the health-related variables in urban population aged 45-72 years according to the distance to green spaces. Table S2. Age-standardized distribution (%) of cardiovascular risk factors in urban population aged 45-72 years according to the distance to green spaces. Table S3. Age-standardized distribution (%) and odds ratios (OR) of chronic diseases in urban population aged 45-72 years according to the distance to green spaces. [file 1476-069X-13-20-S1.doc]

**Additional file 1: Table S1. Age-standardized means (+ SD) of the health-related variables in urban population aged 45-72 years according to the distance to green spaces**

| **Variables** | **Distance to green spaces in tertiles** | | |
| --- | --- | --- | --- |
|  | **1^st^ tertile** | **2^nd^ tertile** | **3^rd^ tertile** |
|  | **N=1694** | **N=1702** | **N=1716** |
| Age, years | 58.5+7.96 | 58.3+7.52 | 57.9+7.62* |
| Systolic BP, mmHg | 139.6+21.1 | 139.3+22.0 | 139.6+21.6 |
| Diastolic BP, mmHg | 90.0+12.1 | 89.6+12.7 | 90.0+12.2 |
| BMI, kgm^2^ | 29.2+5.28 | 29.0+5.28 | 29.1+5.08 |
| Total cholesterol, mmol/L | 5.99+1.14 | 5.97+1.15 | 5.96+1.13 |
| Triglycerides, mmol/L | 1.48+0.97 | 1.46+0.87 | 1.42+0.94 |
| HDL cholesterol, mmol/L | 1.50+0.38 | 1.49+0.38 | 1.53+0.38 |
| LDL cholesterol, mmol/L | 3.82+1.03 | 3.81+1.05 | 3.78+1.02 |
| Fasting glucose, mmol/L | 5.77+1.17 | 5.77+1.17 | 5.78+1.11 |

BMI – body mass index; BP – blood pressure; HDL – high-density lipoproteins; LDL – low-density lipoproteins; * p<0.05, compared to the 1^st^ tertile of distance to green spaces. Distance to green space: the 1^st^ tertile - <347.8 m (high); the 2^nd^ tertile – 347.81 – 629.6 m (moderate); and the 3^rd^ tertile - >629.61 m (low).

**Table S2.** **Age-standardized distribution (%) of cardiovascular risk factors in urban population aged 45-72 years according to the distance to green spaces**

| **Risk factors** | **Distance to green spaces in tertiles** | | | **p value** |
| --- | --- | --- | --- | --- |
|  | **1^st^ tertile N=1694** | **2^nd^ tertile**  **N=1702** | **3^rd^ tertile N=1716** |  |
| **Smoking** |  |  |  | **p=0.006** |
| Regular | 20.0 | 17.0* | 21.1## |  |
| Ex-smoker | 17.4 | 16.3 | 17.9 |  |
| Never | 62.6 | 66.7* | 61.0### |  |
| **Total cholesterol, mmol/L** |  |  |  | p=0.201 |
| < 5.2 | 24.8 | 26.5 | 24.5 |  |
| 5.2-6.19 | 36.5 | 33.9 | 37.7 |  |
| > 6.2 | 38.7 | 39.6 | 37.8 |  |
| **BMI, kg/m^2^** |  |  |  | p=0.717 |
| <25.0 | 22.0 | 22.1 | 21.6 |  |
| 25.0-29.9 | 39.4 | 40.3 | 38.5 |  |
| >30.0 | 38.6 | 37.6 | 39.9 |  |
| **Arterial hypertension, mmHg** |  |  |  | p=0.144 |
| Yes | 66.3 | 63.1 | 64.4 |  |
| No | 33.7 | 36.9 | 35.6 |  |
| **Leisure-time physical activity** |  |  |  | p=0.864 |
| Active | 75.6 | 75.1 | 75.9 |  |
| Inactive | 24.4 | 24.9 | 24.1 |  |
| **Fasting glucose level, mmol/L** |  |  |  | p=0.413 |
| <5.55 | 47.0 | 45.6 | 45.4 |  |
| 5.55-6.99 | 45.8 | 45.4 | 46.0 |  |
| >7.0 | 7.3 | 9.0 | 8.6 |  |
| Yes | 21.8 | 20.9 | 21.4 |  |

BMI –body mass index. * p<0.05, compared to the 2^nd^ tertile of distance to green spaces; ##p<0.01, ###p<0.001, compared to the 2^nd^ tertile of distance to green spaces. Distance to green spaces: the 1^st^ tertile - <347.8 m (high); the 2^nd^ tertile – 347.81 – 629.6 m (moderate); and the 3^rd^ tertile - >629.61 m (low).

Arterial hypertension ”Yes” = mean systolic blood pressure (BP) of at least 140 mm Hg or mean diastolic BP of at least 90 mm Hg, or both, and/or that respondent had been taking antihypertensive drugs during the last two weeks. Arterial hypertension ”No” = systolic BP <140, and diastolic BP <90 mm Hg.

**Table S3. Age-standardized distribution (%) and odds ratios (OR) of chronic diseases in urban population aged 45-72 years according to the distance to green spaces**

| **Distance to green spaces in tertiles** | **Prevalent chronic diseases** | | | **p value** |
| --- | --- | --- | --- | --- |
|  | **No** | **Yes** | **OR* of chronic diseases**  **OR (95% CI)** |  |
| **Men** | **Coronary heart disease:** |  |  | p=0.858 |
| 1^st^ tertile (n=742) | 85.8 | 14.2 | 1 (Reference) |  |
| 2^nd^ tertile (n=734) | 85.0 | 15.0 | 1.12(0.84-1.50) |  |
| 3^rd^ tertile (n=797 | 84.9 | 15.1 | 1.03(0.77-1.39) |  |
|  | **Stroke:** |  |  | p=0.491 |
| 1^st^ tertile (n=742) | 97.8 | 2.2 | 1 (Reference) |  |
| 2^nd^ tertile (n=734) | 98.5 | 1.5 | 0.76(0.35-1.64) |  |
| 3^rd^ tertile (n=797) | 98.4 | 1.6 | 0.82(0.38-1.77) |  |
|  | **Diabetes mellitus:** |  |  | p= 0.254 |
| 1^st^ tertile (n=742) | 92.7 | 7.3 | 1 (Reference) |  |
| 2^nd^ tertile (n=734) | 94.8 | 5.2 | 0.65(0.39-1.09) |  |
| 3^rd^ tertile (n=797) | 93.7 | 6.3 | 0.75(0.45-1.24) |  |
| **Women** | **Coronary heart disease:** |  |  | p=0.286 |
| 1^st^ tertile (n=952) | 81.4 | 18.6 | 1 (Reference) |  |
| 2^nd^ tertile (n=968) | 84.1 | 15.9 | 0.90(0.70-1.14) |  |
| 3^rd^ tertile (n=919) | 82.8 | 17.2 | 0.96(0.75-1.22) |  |
|  | **Stroke:** |  |  | p=0.516 |
| 1^st^ tertile (n=952) | 98.8 | 1.2 | 1 (Reference) |  |
| 2^nd^ tertile (n=968) | 98.6 | 1.4 | 1.34(0.60-3.01) |  |
| 3^rd^ tertile (n=919) | 99.1 | 0.9 | 0.92(0.39-2.19) |  |
|  | **Diabetes mellitus:** |  |  | p=0.390 |
| 1^st^ tertile (n=952) | 92.6 | 7.4 | 1 (Reference) |  |
| 2^nd^ tertile (n=968) | 94.0 | 6.0 | 0.71(0.46-1.11) |  |
| 3^rd^ tertile (n=919) | 93.8 | 6.2 | 0.74(0.48-1.15) |  |
| **Men and women** | **Coronary heart disease:** |  |  | p=0.648 |
| 1^st^ tertile (n=1694) | 83.8 | 16.7 | 1 (Reference) |  |
| 2^nd^ tertile (n=1702) | 84.5 | 15.5 | 0.99(0.82-1.19) |  |
| 3^rd^ tertile (n=1716) | 83.7 | 16.3 | 0.99(0.82-1.20) |  |
|  | **Stroke:** |  |  | p=0.486 |
| 1^st^ tertile (n=1694) | 98.3 | 1.7 | 1 (Reference) |  |
| 2^nd^ tertile (n=1702) | 98.6 | 1.4 | 1.00(0.57-1.73) |  |
| 3^rd^ tertile (n=1716) | 98.8 | 1.2 | 0.85(0.48-1.52) |  |
|  | **Diabetes mellitus:** |  |  | p=0.113 |
| 1^st^ tertile (n=1694) | 92.6 | 7.4 | 1 (Reference) |  |
| 2^nd^ tertile (n=1702) | 94.4 | 5.6 | **0.69(0.49-0.96)** |  |
| 3^rd^ tertile (n=1716) | 93.7 | 6.9 | 0.74(0.54-1.03) |  |

CI – confidence interval. Distance to green space: the 1^st^ tertile - <347.8 m (high); the 2^nd^ tertile – 347.81 – 629.6 m (moderate); and the 3^rd^ tertile - >629.61 m (low). *****adjusted by: age, education, smoking, arterial hypertension, physical activity, total cholesterol level, fasting glucose level, body mass index, and sex (the latter - only in the groups of men and women).
